# The mediating effect of mood spectrum on the relationship between autistic traits and catatonia spectrum

**DOI:** 10.3389/fpsyt.2023.1092193

**Published:** 2023-07-20

**Authors:** Liliana Dell’Osso, Giulia Amatori, Barbara Carpita, Gabriele Massimetti, Benedetta Nardi, Davide Gravina, Francesca Benedetti, Chiara Bonelli, Danila Casagrande, Mario Luciano, Isabella Berardelli, Natascia Brondino, Marianna De Gregorio, Giacomo Deste, Marta Nola, Antonino Reitano, Maria Rosaria Anna Muscatello, Maurizio Pompili, Pierluigi Politi, Antonio Vita, Mario Maj

**Affiliations:** ^1^Department of Clinical and Experimental Medicine, University of Pisa, Pisa, Italy; ^2^Department of Psychiatry, University of Naples “Luigi Vanvitelli”, Naples, Italy; ^3^Department of Neuroscience, Mental Health and Sense Organs, University of Roma “La Sapienza”, Rome, Italy; ^4^Department of Brain and Behavioral Sciences, University of Pavia, Pavia, Italy; ^5^Department of Biomedical and Dental Sciences and Morphofunctional Imaging, University of Messina, Messina, Italy; ^6^Department of Clinical and Experimental Sciences, University of Brescia, Brescia, Italy

**Keywords:** mood disorders, autism, catatonia, autism spectrum, mood spectrum, catatonia spectrum

## Abstract

**Background:**

In the recent years, several studies have shown a correlation between autism spectrum disorder (ASD) and catatonia. It is also known that both conditions are found to be associated with mood disorders. This study aimed to investigate the relationship between autistic traits and catatonic symptoms, as well as the potential mediating role of mood disorder spectrum in the relationship between them.

**Methods:**

The total sample of 514 subjects was composed by four diagnostic groups, composed by patients affected by catatonia (CTN), borderline personality disorder (BPD), major depressive disorder (MDD) and healthy controls (HC). Subjects were assessed with the SCID-5-RV, the Adult Autism Subthreshold Spectrum (AdAS Spectrum) and the Catatonia Spectrum (CS) and the Mood Spectrum Self-Report (MOODS-SR). Statistical analyses included Pearson’s coefficient calculation, multiple linear regression, and mediation analysis.

**Results:**

all the correlations appear to be strongly positive and significant with the strongest coefficient emerging between AdAS Spectrum total score and CS total score (*r* = 0.762, *p* < 0.001). The Mediation Analysis showed that AdAS Spectrum total score showed a significant indirect effect on CS total score through MOODS-SR total score (*b* = 0.168, 95% bootstrapped CI [0.127:0.207]).

**Conclusion:**

The present study highlights the presence of a mediating role of the mood disorder spectrum in the relationship between autistic traits and the catatonia spectrum.

## 1. Introduction

Autism spectrum disorder (ASD) is a neurodevelopmental disorder characterized by impairment in social communication, restricted and repetitive behaviors or interests, and sensory hyper/hyposensitivity. Catatonia, first described in 1874 by Kahlbaum and long confined to the realm of schizophrenia, is now defined by DSM-5-TR^1^ as a severe neuropsychiatric syndrome characterized by three or more symptoms among catalepsy, waxy flexibility, stupor, muteness, negativism, agitation, posturing, stereotypies, mannerisms, grimacing, echolalia, and ecopraxia (1). During the past two decades, the connection between autism spectrum disorder (ASD) and catatonia has been explored by several studies in the scientific literature (2–4).

Complication of ASD with catatonia is not an uncommon occurrence, as already observed by Lorna Wing’s studies on autism (5). A recent systematic review (4) showed that 10.4% of individuals with ASD have catatonia, highlighting a clinical overlap between the two disorders for which various explanations have been suggested: a common alteration in the GABAergic system, of neural circuits (6) or in the size of cerebellar structures (7), as well as a potential genetic linkage arising from susceptibility regions on chromosome 15 (8).

In two prevalence studies (5, 9), catatonia was reported in 12–17% of a large sample of adolescents and young adults having ASD. Numerous clinical features are common to ASD and catatonia, such as mutism and echolalia, stereotyped movements and repetitive behaviors, negativism and arousal. This clinical overlap may be accountable for, on one hand, the tendency to overestimate subthreshold catatonia among autistic subjects and, on the other hand, the under-recognition of catatonic symptoms that first occur in patients with ASD (10). In fact, previous descriptions have mentioned, although anecdotal, that catatonia may develop gradually over the course of autism, usually preceded by isolated manifestations and a slow degradation of functioning, eventually assuming a chronic course (11). One of the few systematic reviews of catatonia among young people with ASD reported that the highest percentage of autism-related cases occur amongst individuals with Asperger’s disorder (AD), rather than classical or atypical autistic patients (12). As it is recognized that a substantial proportion of patients with AD remain undiagnosed during adolescence and adulthood (13), it would be wise to entertain the possibility that they may develop catatonia at some stage in their lives. This situation would demand, from clinicians who work with adults and who are frequently not familiar with the diagnosis of ASD, the capacity to untangle the complex clinical picture of these patients and to properly identify a specific treatment.

In addition to catatonia, mood disorders are also a frequent comorbidity in patients with ASD. In fact, high rates of mood disorders are found in individuals with ASD compared with the general population (14) and Major depression and bipolar disorder are among the most common co-occurring psychiatric diagnoses in autism (15). Co-occurring mood problems significantly impact the well-being and outcomes of people with autism, contributing to reduced quality of life at all developmental phases and to an increase in mortality from suicide (16). Unfortunately, the diagnosis of mood disorder in individuals with ASD can be challenging, both because mood problems can be “obscured” from the main features of autism (17, 18), and also because in autism mood disorders can present with an atypical and autism-specific profile: profiles include reduced or increased restricted and repetitive behaviors and interests, psychomotor agitation, regression, reduced self-care, and severe irritability (19). On the other hand, we know how a severe mood disorder, for example, unrecognized and progressed in its natural history, can result in catatonic manifestations: catatonia is often a presentation of extreme anxiety and depression (20) and the number of catatonic patients among acutely ill psychiatric inpatients varies from 7.6 to 38% (21). Furthermore, higher proportion of catatonic patients have comorbid bipolar disorder (43%) (22).

The aim of the present study is to investigate the relationship between autistic traits and catatonic symptoms, as well as the potential mediating role of the mood disorder spectrum in the relationship between the two, under the hypothesis that mood disorders may play an intermediary role within a psychopathological trajectory strained between the autistic and catatonic spectrum.

## 2. Materials and methods

Research data were collected between November 2021 and January 2022 at six Italian university departments of psychiatry, coordinated by the University of Pisa: University of Campania “Luigi Vanvitelli,” University of Pavia, University of Messina, University La Sapienza of Rome, and University of Brescia.

### 2.1. Study sample and procedures

The total sample was composed of 514 subjects spread across four diagnostic groups, all of which were evaluated in accordance with the DSM-5 diagnostic criteria. The exclusion criteria included: age below 18 years, language or intellectual impairments affecting the capability to conduct assessments, mental disability, low cooperation ability, and ongoing psychotic symptoms. In particular, the four groups were defined as follows: 106 outpatients with at least 3 symptomatic criteria for catatonia (CTN); 105 outpatients with a diagnosis of borderline personality disorder (BPD); 147 outpatients with a diagnosis of major depressive disorder (MDD); and 156 healthy controls with no actual or lifetime mental disorders (HC) recruited from health care and paramedical personnel. All subjects were aged between 18 and 60 years and signed a written informed consent. The Structured Clinical Interview for DSM-5, Research Version (SCID-5-RV) (23) was administered to validate the diagnoses of BPD and MDD as well as the absence of mental disorders among CTLs. The study was carried out in conformity with the Declaration of Helsinki. The Ethics Committee of the Azienda Ospedaliero-Universitaria di Pisa approved all selection and assessment procedures. The eligible subjects supplied written informed consent after receiving a complete explanation of the study and being provided with an opportunity to pose questions. Subjects were not remunerated for their participation in accordance with Italian law.

### 2.2. Measures

Assessment procedures included the SCID-5-RV (23), the Adult Autism Subthreshold Spectrum (AdAS Spectrum), the Catatonia Spectrum (CS) and the Mood Spectrum Self-Report Questionnaire (MOOS-SR) were carried by psychiatrists who were trained and certified in the use of the study instruments.

#### 2.2.1. The adult autism subthreshold Spectrum

The Adult Autism Subthreshold Spectrum (AdAS Spectrum) (24) is a questionnaire designed to assess not only overt ASD, but also the broader spectrum of subthreshold autism, in individuals without cognitive impairment and language disorders across the lifespan. It enables the evaluation of a wide range of clinical and nonclinical traits, typical and atypical manifestations, including a number of gender-specific features. The tool is consists of dichotomous questions, clustered into seven domains: Infancy/Adolescence, Verbal Communication, Nonverbal Communication, Empathy, Inflexibility and Adherence to Routine, Restricted Interests and Rumination, and Hyper-ipo reactivity to sensory input. In the validation study. The AdAS Spectrum questionnaire showed excellent reliability and strong convergent validity with other scales used in this field, such as the Autism-Spectrum Quotient Test (25) and the e Ritvo Autism and Asperger’s Diagnostic Scale 14-item version (26). The AdAS Spectrum has been employed within several studies focusing on the autism spectrum in both clinical and nonclinical settings in recent years (27–35). The cut-off scores to identify subjects with full-blown ASD and significant autistic traits corresponded to 70 and 43, respectively (36).

#### 2.2.2. The catatonia spectrum

The Catatonia Spectrum (CS) is a self-assessment questionnaire that evaluates nuclear, subthreshold, atypical and partial manifestations of the Catatonia Spectrum, referred across the lifespan and clustered into domains. The CS is composed of 74 items and divided into 8 domains: (1) Psychomotor activity (Stupor); (2) Verbal response (Mutism); (3) Repetitive movements (Stereotypes); (4) Artificial expressions and actions (Mannerisms); (5) Oppositivity or poor response to stimuli (Negativism); (6) Response to instructions given from outside (Automatic obedience); (7) Automatisms; (8) Impulsivity. For each item there is a dichotomous answer “Yes” and “No.”

In the validation study (37), the CS questionnaire showed excellent internal consistency and test–retest reliability and strong convergent validity with alternative dimensional measures of catatonia, such as the Bush-Francis Catatonia Rating Scalee (37) and the Bush-Francis Catatonia Screening Instrument (38).

#### 2.2.3. The mood spectrum self-report

The mood Spectrum self-report (MOODS-SR) is a questionnaire designed to assess the broad spectrum of mood symptoms, including suicidal ideation and behavior, and temperamental characteristics across the life span. Being a dimensional instrument, it is intended to identify even mild and subthreshold manifestations, which may present with prodromal, residual or atypical clinical pictures. Items are grouped into seven domains, three assessing the manic/hypomanic pole and three assessing the depressive pole in the dimensions of cognition, energy, and mood, respectively, together with an additional domain exploring rhythmicity and vegetative functions (39).

### 2.3. Statistical analysis

Comparisons between CS, AdAS Spectrum and MOOD-SR total scores between the four diagnostic groups were performed by Analysis of Variance (ANOVA).

To evaluate the association between the total AdAS Spectrum, MOODS-SR and the CS total scores, we calculated the Pearson correlation coefficients. Subsequently, to evaluate the confounding effects of the independent variables (AdAS Spectrum and MOODS-SR total scores) associated with the dependent variable (CS total score) and to verify whether AdAS Spectrum and MOODS-SR are good predictors of the CS score, a multiple linear regression was utilized. Since the two independent variables were significantly associated with the CS total score, we then performed a mediation analysis providing AdAS Spectrum total score as predictor, CS total score as dependent variable and MOODS-SR total score as mediator. The Hayes’s PROCESS tool was utilized; bootstrap confidence intervals for not standardized and standardized indirect effect were computed.

All analyses were performed using SPSS version 26 (IBM Corp 2019) (40).

## 3. Results

The catatonia group included subjects with a mean age of 43.13 years (± 12.64), 44 (41.5%) males and 62 (58.05%) females. The MDD subjects had a mean age of 45.39 (±12.46) years and consisted of 54 (36.7%) males and 93 (63.13%) females. The group of BDP subjects had a mean age of 36.65 (±14.09) years and consisted of 31 (29.5%) males and 74 (70.5%) females. The group of HC had a mean age of 34.48 (±10.24) years and consisted of 70 (44.9%) males and 86 (55.1%) females. The comparisons between CS, AdAS Spectrum and MOOD-SR total scores between the four diagnostic groups are reported in Table 1.

**Table 1:** Comparison of CS, AdAS Spectrum and MOOD-SR total scores between the four diagnostic groups.

	CS total score	AdAS Spectrum total score	MOODS-SR total score	*p*
(a) CTN (mean + SD)	36,273 (16,335)	58,755 (27,154)	62,368 (25,884)	< 0.001
(b) BPD (mean + SD)	39,190 (14,91,843)	74,314 (27,349)	83,305 (25,563)	< 0.001
(c) MDD (mean + SD)	30,244 (16,851)	54,905 (31,743)	64,612 (29,942)	< 0.001
(d) HC (mean + SD)	19.026 (12.026)	31.237 (21.026)	31.980 (21.224)	< 0.001
F (degrees of freedom)	46.997 (3)	57.325 (3)	90.478 (3)	–
*Post hoc*	a > c, d; b > c, d; c > d	b > a, c, d; c > d	b > a, c, d; c > d	< 0.001

All the correlations appeared to be strongly positive and significant: between AdAS Spectrum and MOOD-SR total score (*r* = 0.724, *p* < 0.001), between MOOD-SR and CS total score (*r* = 0.755, *p* < 0.001) and, to the greatest extent, between AdAS Spectrum total score and CS total score (*r* = 0.762, *p* < 0.001).

A multiple linear regression was performed using CS total score as dependent variable and AdAS Spectrum and MOODS-SR total scores as independent variables. Results are shown in Table 2. The two independent variables significantly predict the level of CS, with the AdAS-Spectrum showing the highest standardized regression coefficient (*β* = 0.453).

**Table 2:** Multiple linear regression with CS total score as dependent variable.

	B (SE)	*β*	CI 95%	*t*	*p*
*K*	3.848 (0.926)		2.028–5.668	4.154	< 0.001
AdAS Spectrum	0.246 (0.020)	0.453	0.207–0.286	12.240	< 0.001
MOODS-SR	0.226 (0.020)	0.426	0.188–0.265	11.520	< 0.001

*R*^2^ = 0.667, Adjusted *R*^2^ = 0.666.

**Figure 1:**
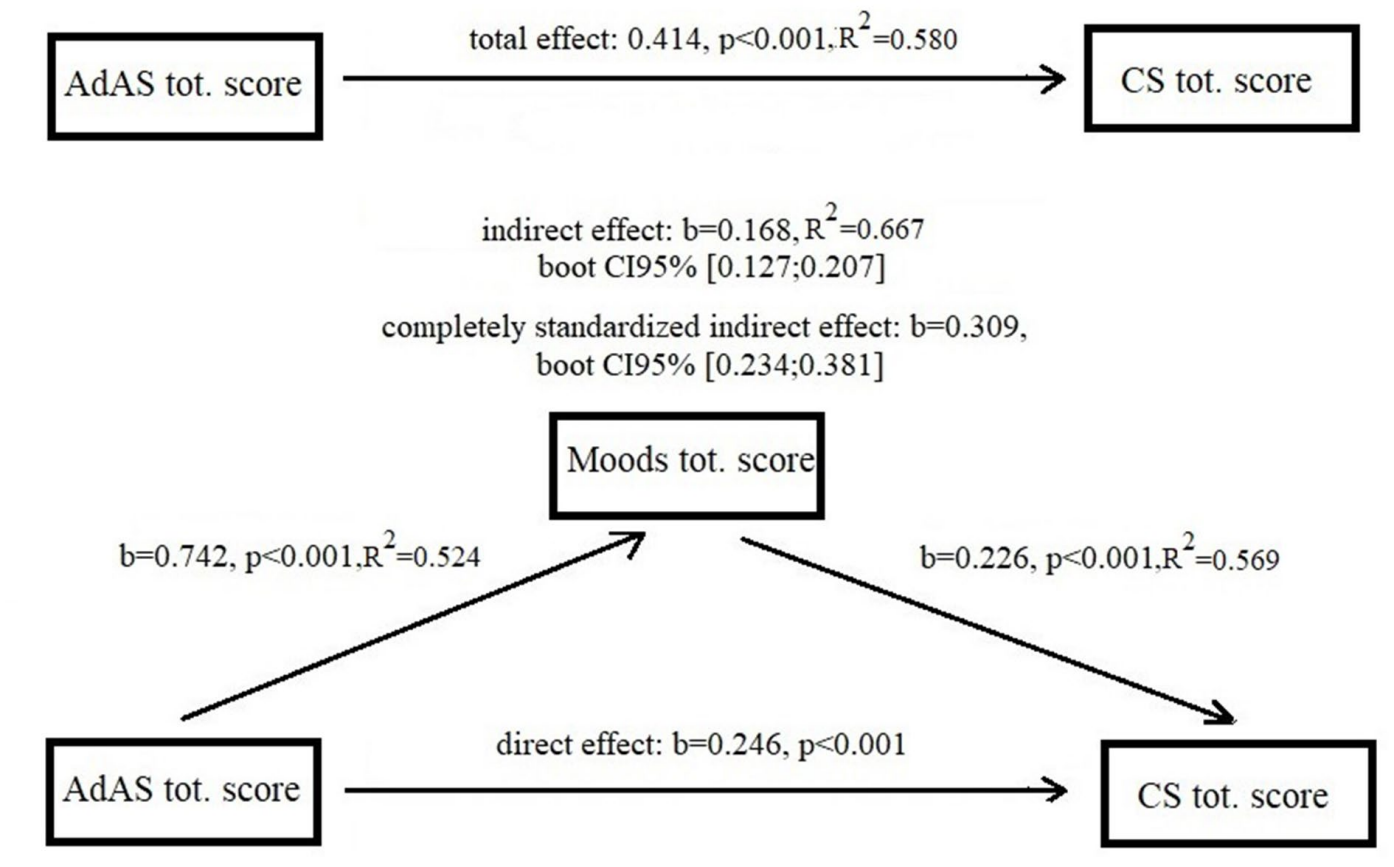
Mediation analysis results.

The Mediation Analysis (Figure 1) showed that total and direct effect od AdAS Spectrum total score on CS total score were statistically significant (total effect = 1.414, *p* < 0.001, direct effect = 0.246, *p* < 0.001). AdAS Spectrum total score also showed a significant indirect effect on CS total score through MOODS-SR total score (*b* = 0.168, 95% bootstrapped CI [0.127:0.207]). The standardized indirect effect (or index of Mediation) was *b* = 0.309 with 95% bootstrapped CI [0.234:0.381].

## 4. Discussion

The results of the current study suggest a strong correlation between the autism spectrum (autistic traits and signs, symptoms, and behavioral manifestations of ASD) and the catatonia spectrum (nuclear, subthreshold, atypical, and partial manifestations), in accordance with prior literature on the correlation between ASD and catatonia.

The finding, in the group of patients with BPD, of high mean scores on questionnaires investigating the autistic (AdAS Spectrum), mood (MOOD-SR) and catatonic spectrum (CS) could be explained by considering that borderline personality disorder represents a mental disorder of extreme severity: 80% of patients with BPD have suicidal behaviors or suicide attempts, and 4 to 9% of them die by suicide (41, 42). For these reasons, the management of borderline personality disorder represents one of the greatest challenges in modern psychiatry. These findings are in agreement with previous studies that support the existence of a psychopathological trajectory in which the various mental disorders would lie along a continuum of severity and in which BPD would represent the point of maximum severity (37, 43).

The results also reveal an intermediary role of the mood disorder spectrum in the relationship between autistic traits and the catatonia spectrum.

As a supplement to the categorical approach currently used by our nosographic system, in recent decades numerous studies have hypothesized that mental disorders can be better framed as parts of a continuum, having as their common basis an alteration in neurodevelopment. The “neurodevelopmental continuum” represents a concept supported by a great deal of empirical evidence, and the conceptual core underlying this model is that an alteration in brain development, the expression of which would be determined by the relationships between genetic and environmental factors, may form the common basis of different types of mental disorders (44).

In addition, the DSM-5 does not fully account for the broader spectrum of subthreshold manifestations distributed along a continuum in the general population, as well as the broad occurrence of autism-like traits in the clinical population of individuals with other mental disorders (24, 27). Subthreshold autistic traits were first noticed by studies among unaffected first-degree family members of probands with ASD (27, 45, 46). Therefore, their heterogeneous distribution in the general population and in specific high-risk groups, has been demonstrated (47–50). Autistic traits appears to be highly prevalent in a wide variety of clinical groups, included in patients with mood disorders, where it may represent a specific risk factor for suicidal ideation and behaviors (51–53).

Regarding possible limitations in the present study, it is necessary to mention how the use of self-report tools returns a less accurate assessment than direct evaluation of a clinician. On the other hand, the use of spectrum questionnaires enabled to assess the examined mental disorders in the full range of their manifestations, from subthreshold symptoms to overt manifestations, showing how mood disorders could mediate the progression of a psychopathological trajectory from an autistic vulnerability substrate toward catatonia, a clinical syndrome very often associated with severe mental disorders.

## 5. Conclusion

The results of the present study show a strong correlation between autistic traits and the manifestations of catatonia spectrum.

Moreover, the findings highlight a mediating role of the mood disorder spectrum in the relationship between autistic traits and the catatonia spectrum.

## Data availability statement

The raw data supporting the conclusions of this article will be made available by the authors, without undue reservation.

## Ethics statement

The studies involving human participants were reviewed and approved by the Comitato Etico Azienda Ospedaliero Universitaria Pisana. The patients/participants provided their written informed consent to participate in this study.

## Author contributions

LD’O conceived and revised the work. GM done the statistical analysis. GA and LD’O drafted the manuscript. LD’O and BC revised the manuscript. All authors collected the data processed in the study and provided approval of the version to be published.

## Conflict of interest

The authors declare that the research was conducted in the absence of any commercial or financial relationships that could be construed as a potential conflict of interest.

## Publisher’s note

All claims expressed in this article are solely those of the authors and do not necessarily represent those of their affiliated organizations, or those of the publisher, the editors and the reviewers. Any product that may be evaluated in this article, or claim that may be made by its manufacturer, is not guaranteed or endorsed by the publisher.
